# Temporal trends in glucocorticoids and hydroxychloroquine for treatment of systemic lupus erythematosus in Sweden

**DOI:** 10.1093/rheumatology/keaf192

**Published:** 2025-04-10

**Authors:** Annica Dominicus, Arthur Mageau, Ngoc V Nguyen, Karin Blomkvist Sporre, Elisabet Svenungsson, Elizabeth V Arkema

**Affiliations:** Clinical Epidemiology Division, Department of Medicine Solna, Karolinska Institutet, Karolinska University Hospital, Stockholm, Sweden; Clinical Epidemiology Division, Department of Medicine Solna, Karolinska Institutet, Karolinska University Hospital, Stockholm, Sweden; Internal Medicine Department, Hôpital Bichat-Claude Bernard, AP-HP, Université Paris Cité, Paris, France; Clinical Epidemiology Division, Department of Medicine Solna, Karolinska Institutet, Karolinska University Hospital, Stockholm, Sweden; Division of Rheumatology, Department of Medicine Solna, Karolinska Institutet, Karolinska University Hospital, Stockholm, Sweden; Division of Rheumatology, Department of Medicine Solna, Karolinska Institutet, Karolinska University Hospital, Stockholm, Sweden; Clinical Epidemiology Division, Department of Medicine Solna, Karolinska Institutet, Karolinska University Hospital, Stockholm, Sweden

**Keywords:** systemic lupus erythematosus, glucocorticoids, hydroxychloroquine

## Abstract

**Objectives:**

It is unknown to what extent updated treatment recommendations regarding glucocorticoids (GC) and hydroxychloroquine (HCQ) for patients with systemic lupus erythematosus (SLE) have been incorporated into clinical practice. Based on filled dispensations we examined treatment patterns the first 5 years after SLE diagnosis in Sweden, trends over time and relationship to patient characteristics.

**Methods:**

A cohort of patients with newly diagnosed SLE between 2005 and 2021 with information on drug dispensations, hospitalizations, specialized outpatient visits and patient characteristics were identified through a linkage of Swedish population registers (*n* = 3891, 83% females, mean age 48.8). Treatment patterns, including accumulated exposure to GC and HCQ and combinations of treatments, were investigated in relation to year of diagnosis and patient characteristics using visualizations, logistic regression and quantile regression analysis.

**Results:**

The proportion of SLE patients treated with GC during the first year after diagnosis was 68.3% over the study period. For the fifth year it decreased from 54.1% to 46.3%. The median decrease in 5-year cumulative GC dose attributable to calendar year was 753 mg (90% CI: 1560 mg decrease, 106 mg increase) with a more pronounced trend towards fewer patients on the highest exposure levels. The median increase in proportion of days covered with HCQ during 5 years was 28.6% (90% CI: 21.9%, 36.2%).

**Conclusion:**

The modest reduction of GC exposure and substantial increase in HCQ coverage over time aligns with changes in recommendations for SLE management. However, treatment optimization and continued efforts to raise awareness remain essential to ensure equal care and improve clinical outcomes.

## Graphical abstract

**Figure:**
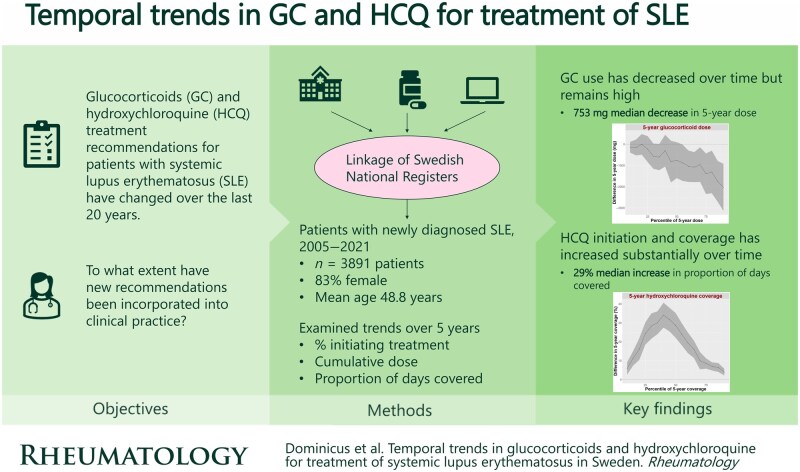
Graphical abstract showing the study results that glucocorticoid use has decreased over time but remains high, while hydroxychloroquine initiation and coverage have increased substantially over the study period.

Rheumatology key messages

- Glucocorticoid usage over the first 5 years after SLE diagnosis has decreased over time but remains high.
- Hydroxychloroquine use has increased substantially, in terms of both early initiation and coverage during the first 5 years after SLE diagnosis.
- Observed associations between glucocorticoid and hydroxychloroquine use and patient characteristics and hospitals where SLE diagnosis was received call for continued work to ensure equal care.

## Introduction

Systemic lupus erythematosus (SLE) is a heterogeneous disease with a variable course involving multiple organs and requiring different and dynamic treatments [1]. Treatment guidelines have been updated over time as more evidence on treatment effectiveness and safety has accumulated. Acknowledging the increased risk of organ damage associated with long-term use of glucocorticoids (GC), the 2019 update of EULAR guidelines [2] recommended a maximum daily dose of 7.5 mg prednisolone-equivalent GC, which was recently updated to a maximum of 5 mg [3]. In general, GC should only be used during periods of increased disease activity and, when possible, be reduced or discontinued. Hydroxychloroquine (HCQ) is recommended for all patients with SLE, considering the individual’s risk for flares and retinal toxicity [2–5]. However, it is not well understood to what extent new guidelines have led to changes in SLE treatment in practice. Immunosuppressants (IS) and more recently biologic agents are also important options to be considered to control the disease and facilitate GC tapering/discontinuation.

Treatment patterns for SLE patients are often complex, reflecting changes in disease manifestations, flares and the overarching treatment target of balancing risk with benefit for each individual. Previous studies have described treatment patterns in SLE populations [6–9], but not specifically evaluated temporal trends. In reports of temporal trends, long-term use of all common treatments have not been addressed [10].

To fully evaluate the safety profiles of medications in SLE, measures of medication exposure over longer time periods accounting for time-varying dosing patterns have to be well understood and evaluated in relation to adverse outcomes. GC exposure has been recognized as a major factor contributing to damage in patients with SLE [11, 12]. Not surprisingly, results have been inconsistent since GC exposure is difficult to quantify as dosages can vary greatly over short periods of time and side effects make non-compliance to prescribed dosages common. Often GC exposure is simplified as ‘any use’, current daily dose or at best cumulative dose based on prescriptions in medical files. These reports may not capture the different aspects of GC exposure (duration, timing and dosing) which are likely important for different types of adverse outcomes [11, 13]. Furthermore, the indication for GCs is active disease, making it difficult to disentangle which is more harmful, high disease activity or GC use. For these reasons we need more reliable measures of GC exposure, accounting for time-varying dosing patterns that can track use over longer time periods in large cohorts. Further, to address the role of long-term medication exposure for the risk of adverse outcomes in observational studies, confounding by indication has to be accounted for, requiring a good understanding of the relationship between patient characteristics and medication patterns. Previous studies have reported associations between SLE treatments and treatment centre, age, race/ethnicity, sex, comorbidities, health-care utilization, and disease duration, activity and severity [6–9, 14]. In these studies, potential changes in clinical practice over time have not been addressed.

Using a linkage of nationwide population-based Swedish registers over two decades, we aimed to investigate SLE treatment patterns and variation across time and patient characteristics. We were interested in quantifying accumulated exposure accounting for dose and duration to assess whether recommendations to keep GC at a minimum are reflected in practice and to what extent continuous treatment with HCQ has increased.

## Methods

### Study population

In this cohort study we included adults (≥18 years old) with newly diagnosed SLE and identified their treatments and patient characteristics from several Swedish national registers. Data from the National Patient Register (NPR) was used to identify all unique individuals with at least two visits listing an International Classification of Diseases code for SLE (ICD-10 M32.1, M32.8 and M32.9), occurring within 1 year and requiring at least one visit with a specialist (clinics/departments that typically diagnose SLE, including rheumatology, dermatology, nephrology, internal medicine and paediatrics) [15]. The NPR includes inpatient hospitalizations since 1964 (nationwide since 1987) and specialized outpatient visits since 2001. Incident SLE was identified by restricting the population to individuals with their first SLE-coded visit between 1 July 2005 and 1 September 2021. This allows for a time window of 4.5 years to detect and exclude prevalent cases. To ensure that individuals had incident SLE, patients were required to be living in Sweden for at least 2 years prior to SLE diagnosis. With focus on evaluation of treatments, patients were required to have at least 1 year of follow-up data. The study was approved by the Swedish Ethical Review Authority (DNR 2021-01148). Participant consent was not required for this register-based study with pseudonymized data in accordance with Swedish law.

### SLE treatments

The Prescribed Drug Register (PDR) contains all dispensed prescribed drugs at pharmacies in Sweden since July 2005. We collected information on dispensations of GC, HCQ and IS including azathioprine (AZA), methotrexate (MTX), mycophenolate mofetil (MMF), cyclophosphamide, leflunomide, tacrolimus and ciclosporin from the PDR (July 2005 through January 2024; treatment codes provided in Supplementary Table S1, available at *Rheumatology* online). Treatment with GC, HCQ and IS was assessed yearly and defined as at least one dispensation during the 1-year intervals calculated from the date of first SLE diagnosis.

Cumulative GC dose was derived from information on the defined daily dose (DDD) and number of DDDs of filled prescriptions. GC dose was transformed to prednisolone equivalent dose (transformation factors described in Supplementary Table S1, available at *Rheumatology* online). In calculations of days covered from a HCQ dispensation the number of days was calculated as the number of packages × 100 (100 pills of 200 mg per package). If a new dispensation occurred within this time frame, the first dispensation was assumed to be consumed completely prior to the new dispensation, and the new dispensation assumed to cover the days following the new dispensation date. In cases with a single dispensation of more than one package, each package was assumed to cover 75 days (corresponding to 300 mg/day). This definition captures the variability in dosages that exist in clinical practice, with a maximum recommended dose of 5 mg/kg real body weight [2, 3, 5]. Days covered were truncated at end of follow-up. The proportion of days covered (PDC) was calculated as the number of days covered divided by number of days of follow-up in the period of interest. Cumulative GC dose and HCQ PDC were evaluated yearly as well as over the first 5 years after SLE diagnosis. Summarizing exposure over 5 years and conducting analyses of various percentiles of the distribution of accumulated exposure allowed for an evaluation of long-term exposure without making any assumptions regarding prescribed daily doses.

### Covariates

Calendar year of SLE diagnosis, the covariate of main interest in the analysis of temporal trends in SLE treatment patterns, was categorized into periods 2005–2008, 2009–2012, 2013–2016 and 2017–2021. Age at diagnosis, sex and country of birth (Nordic, non-Nordic) were retrieved from the Total Population Register. Type of hospital where SLE diagnosis was received (university/other), days of hospitalization and number of specialized outpatient visits during the year preceding SLE diagnosis were retrieved from the NPR. Educational level (highest education achieved) was collected from the Education Register.

### Statistical analysis

The proportion of patients with any dispensation of GC or HCQ was estimated for year 1 and 5 after SLE diagnosis for the different calendar cohorts and temporal trends were analysed using logistic regression models including calendar year as a covariate and adjusting for all covariates listed above. Yearly dispensed GC dose and HCQ PDC were described in alluvial plots stratified on year of SLE diagnosis.

Further, cumulative GC dose and HCQ PDC for the first 5 years after SLE diagnosis were analysed for patients with at least one dispensation during that period. The overall temporal trend was tested through Wilcoxon two-sample tests comparing the calendar cohorts. Association with calendar time was further evaluated through quantile regression [16] adjusting for patient characteristics (age, sex, educational level, country of birth, hospital for SLE diagnosis [university/other], days of hospitalizations and number of specialized outpatient visits during the year preceding the first SLE diagnosis). In these analyses we examined the full distribution of the 5-year medication usage through analyses of all percentiles in the range 10–90% (in 5% steps). This allows for an assessment of temporal trends not only of the median (50th percentile) exposure, but also to what extent very high or very low medication usage has changed over time. Estimates were reported together with 90% confidence intervals (CIs) obtained by bootstrap methods.

All analyses were run in R version 4.4.1 (R Foundation for Statistical Computing, Vienna, Austria) using quantreg package v5.98 for quantile regression analyses [17].

## Results

### Incident SLE in Sweden 2005–2021

We identified 3891 individuals with their first SLE diagnosis 2005–2021 fulfilling the inclusion criteria and having at least 1 year of follow-up, with mean age at diagnosis 48.8 (s.d. 18.1), 82.5% female. Patient characteristics of incident SLE cases changed slightly over the observed time period, with higher educational levels, more patients with non-Nordic country of birth and fewer days of hospitalizations the year preceding SLE diagnosis (Table 1). Overall, the mean follow-up time was 8.5 years (s.d. 4.6, range 1.0–17.3).

**Table 1.** Characteristics of patients with newly diagnosed SLE by year of SLE diagnosis

	SLE diagnosis				
	2005–2008 (*n* = 835)	2009–2012 (*n* = 995)	2013–2016 (*n* = 967)	2017–2021 (*n* = 1094)	Total (*n* = 3891)
Sex, *n* (%)					
Female	699 (83.7)	828 (83.2)	791 (81.8)	893 (81.6)	3211 (82.5)
Male	136 (16.3)	167 (16.8)	176 (18.2)	201 (18.4)	680 (17.5)
Age at SLE diagnosis, years					
Mean (s.d.)	48.7 (17.7)	48.8 (17.6)	49.1 (18.3)	48.8 (18.6)	48.8 (18.1)
Median [min, max]	49.0 [18.0, 94.0]	49.0 [18.0, 90.0]	48.0 [18.0, 95.0]	47.0 [18.0, 96.0]	48.0 [18.0, 96.0]
Age at SLE diagnosis, *n* (%)					
18–39 years	281 (33.7)	341 (34.3)	330 (34.1)	411 (37.6)	1363 (35.0)
40–59 years	304 (36.4)	343 (34.5)	310 (32.1)	328 (30.0)	1285 (33.0)
≥60 years	250 (29.9)	311 (31.3)	327 (33.8)	355 (32.4)	1243 (31.9)
Country of birth, *n* (%)					
Nordic	733 (87.8)	846 (85.0)	778 (80.5)	876 (80.1)	3233 (83.1)
Non-Nordic	90 (10.8)	139 (14.0)	171 (17.7)	205 (18.7)	605 (15.5)
Missing	12 (1.4)	10 (1.0)	18 (1.9)	13 (1.2)	53 (1.4)
SLE diagnosis, *n* (%)					
University hospital	415 (49.7)	491 (49.3)	492 (50.9)	505 (46.2)	1903 (48.9)
Other hospital	420 (50.3)	504 (50.7)	475 (49.1)	589 (53.8)	1988 (51.1)
Educational level, *n* (%)					
0–9 years	221 (26.5)	245 (24.6)	205 (21.2)	185 (16.9)	856 (22.0)
10–12 years	368 (44.1)	431 (43.3)	426 (44.1)	472 (43.1)	1697 (43.6)
≥13 years	245 (29.3)	313 (31.5)	327 (33.8)	427 (39.0)	1312 (33.7)
Missing	1 (0.1)	6 (0.6)	9 (0.9)	10 (0.9)	26 (0.7)
Hospitalization^a^, *n* (%)					
0 days	523 (62.6)	612 (61.5)	582 (60.2)	746 (68.2)	2463 (63.3)
1–7 days	165 (19.8)	205 (20.6)	208 (21.5)	191 (17.5)	769 (19.8)
>7 days	147 (17.6)	178 (17.9)	177 (18.3)	157 (14.4)	659 (16.9)
Outpatient visits^a^, *n* (%)					
0–2	370 (44.3)	389 (39.1)	331 (34.2)	373 (34.1)	1463 (37.6)
3–6	280 (33.5)	346 (34.8)	341 (35.3)	384 (35.1)	1351 (34.7)
>6	185 (22.2)	260 (26.1)	295 (30.5)	337 (30.8)	1077 (27.7)

a 1 year preceding SLE diagnosis.

In this cohort, 83.7% received GC, 76.9% received HCQ and 53.7% received an IS at least once during follow-up. Analysis of treatment patterns over the first 5 years after diagnosis was based on patients diagnosed 2005–2016 (*n* = 2797), of which 2590 had at least 5 years of follow-up. Among these, 87.1% received GC, 76.9% received HCQ and 57.4% received an IS at least once during these first 5 years.

### Temporal trends in use of GC and HCQ

Use of GC at least once in the first year after SLE diagnosis increased for those diagnosed 2009–2016 compared with 2005–2008, but returned to the same level in 2017–2021 (68.3%; Table 2). However, GC use during the fifth year after SLE diagnosis decreased over time from 54.1% in the 2005–2008 cohort to 46.3% in the 2013–2016 cohort (adjusted OR = 0.71, 95%CI 0.58–0.86, *P* = 0.001; Table 2). Results from the fully adjusted model (Supplementary Table S2, available at *Rheumatology* online) show an increased probability of receiving GC in the fifth year with older age, and high healthcare utilization the year preceding SLE diagnosis.

**Table 2.** Association between glucocorticoid and hydroxychloroquine use during first and fifth year after SLE diagnosis and year of diagnosis

	SLE diagnosis			
	2005–2008	2009–2012	2013–2016	2017–2021
Year 1: patients with ≥1 year follow-up				
*n*	835	995	967	1094
Glucocorticoid use				
*n* (%)	568 (68.0)	727 (73.1)	707 (73.1)	747 (68.3)
OR (95% CI)^a^	Ref.	1.23 (1.00, 1.52)	1.21 (0.98, 1.50)	0.98 (0.80, 1.21)
*P*		0.048	0.070	0.862
Hydroxychloroquine use				
*n* (%)	369 (44.2)	636 (63.9)	706 (73.0)	877 (80.2)
OR (95% CI)^a^	Ref.	2.46 (2.02, 3.00)	3.90 (3.16, 4.82 )	5.53 (4.44, 6.91)
*P*		<0.001	<0.001	<0.001
Year 5: patients with ≥5 years follow-up				
*n*	771	922	897	—
Glucocorticoid use				
*n* (%)	417 (54.1)	457 (49.6)	415 (46.3)	—
OR (95% CI)^a^	Ref.	0.81 (0.66, 0.98)	0.71 (0.58, 0.86)	—
*P*		0.032	0.001	
Hydroxychloroquine use				
*n* (%)	314 (40.7)	472 (51.2)	539 (60.1)	—
OR (95% CI)^a^	Ref.	1.61 (1.32, 1.96)	2.32 (1.89, 2.85)	—
*P*		<0.001	<0.001	

a Odds ratio (OR) comparing different calendar cohorts and the odds of having at least one dispensation during first and fifth year after SLE diagnosis (separate analyses of first and fifth year, and of glucocorticoids and hydroxychloroquine; reference SLE diagnosis 2005–2008). Estimated from logistic regression analysis adjusted for age, sex, educational level, country of birth, type of hospital at SLE diagnosis (university/other), number of hospitalization days, and specialized outpatient visits during the year preceding SLE diagnosis.

**Figure 1.**
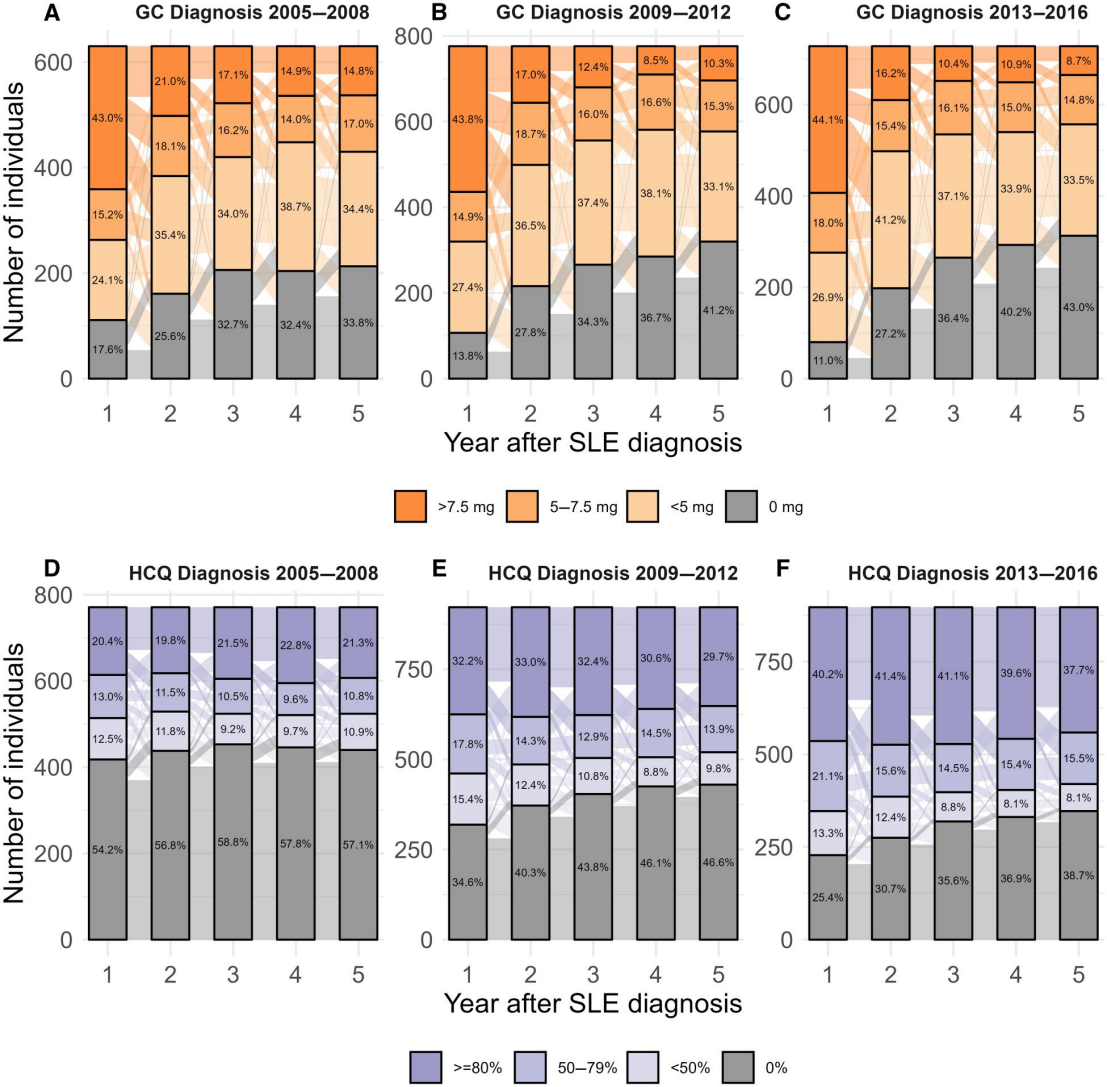
Average daily dose of glucocorticoids (GC; prednisolone equivalent; **A**), and hydroxychloroquine proportion of days covered (HCQ; **B**) during each of the first 5 years after SLE diagnosis

The highest proportion of patients with average daily GC dose >7.5 mg was during the first year after diagnosis, with tapering of yearly doses seen by a downward flow of individuals from one year to the next in Fig. 1A. This suggests that the average daily dose remains on the same level or decreases over time for the majority of patients who are initially treated with GCs, which has not changed over time.

With respect to HCQ, the proportion of patients with at least one dispensation during the first year after SLE diagnosis increased from 44.2% in 2005–2008 to 80.2% in 2017–2021 (Table 2). During the fifth year, the proportion was 40.7% and 60.1%, for the first and last calendar cohorts, respectively. The increased probability of being on HCQ during the first and fifth year after diagnosis associated with calendar time was also clear after adjustment for patient characteristics (Table 2). Association with other patient characteristics is shown in Supplementary Table S3, available at *Rheumatology* online. The HCQ coverage had also increased substantially, with ∼40% of patients having at least 80% HCQ PDC from the first through the fifth year after diagnosis for the last calendar cohort (Fig. 1B).

### Temporal trends in cumulative exposure to GC and HCQ

**Figure 2.**
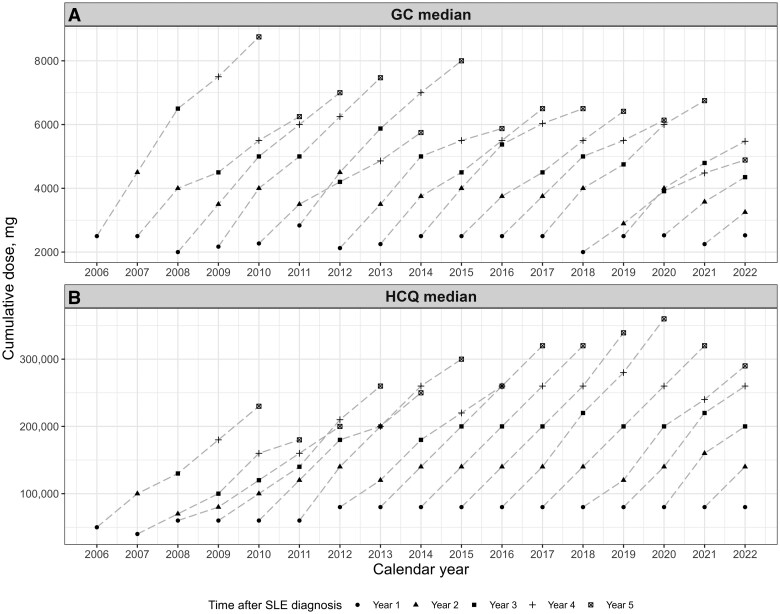
Cumulative dose of glucocorticoid (GC; **A**) and hydroxychloroquine (HCQ; **B**), over the first 5 years after SLE diagnosis over calendar time

Fig. 2 shows cumulative doses of GC (Fig. 2A) and HCQ (Fig. 2B) over calendar time, up to 5 years after SLE diagnosis. The median 5-year cumulative GC dose among patients with at least 5 years’ follow-up (diagnosis in 2005–2016) and receiving GC at least once was 7000 mg, ranging from 5789 mg (diagnosis year 2009) to 9500 mg (diagnosis year 2005).

Among patients treated with GC at least once during the first 5 years after diagnosis, the median (and interquartile range) cumulative GC dose over this period was 7500 (3000–12 000) mg, 7000 (2274–11 606) mg and 6500 (2650–11 000) mg for calendar cohorts 2005–2008, 2009–2012, and 2013–2016, respectively. In crude group-wise comparisons the decrease was close to statistically significant (*P* = 0.077 and *P* = 0.050 for Wilcoxon two-sample test comparing 2009–2012 and 2013–2016 *vs* 2005–2008, respectively).

**Figure 3.**
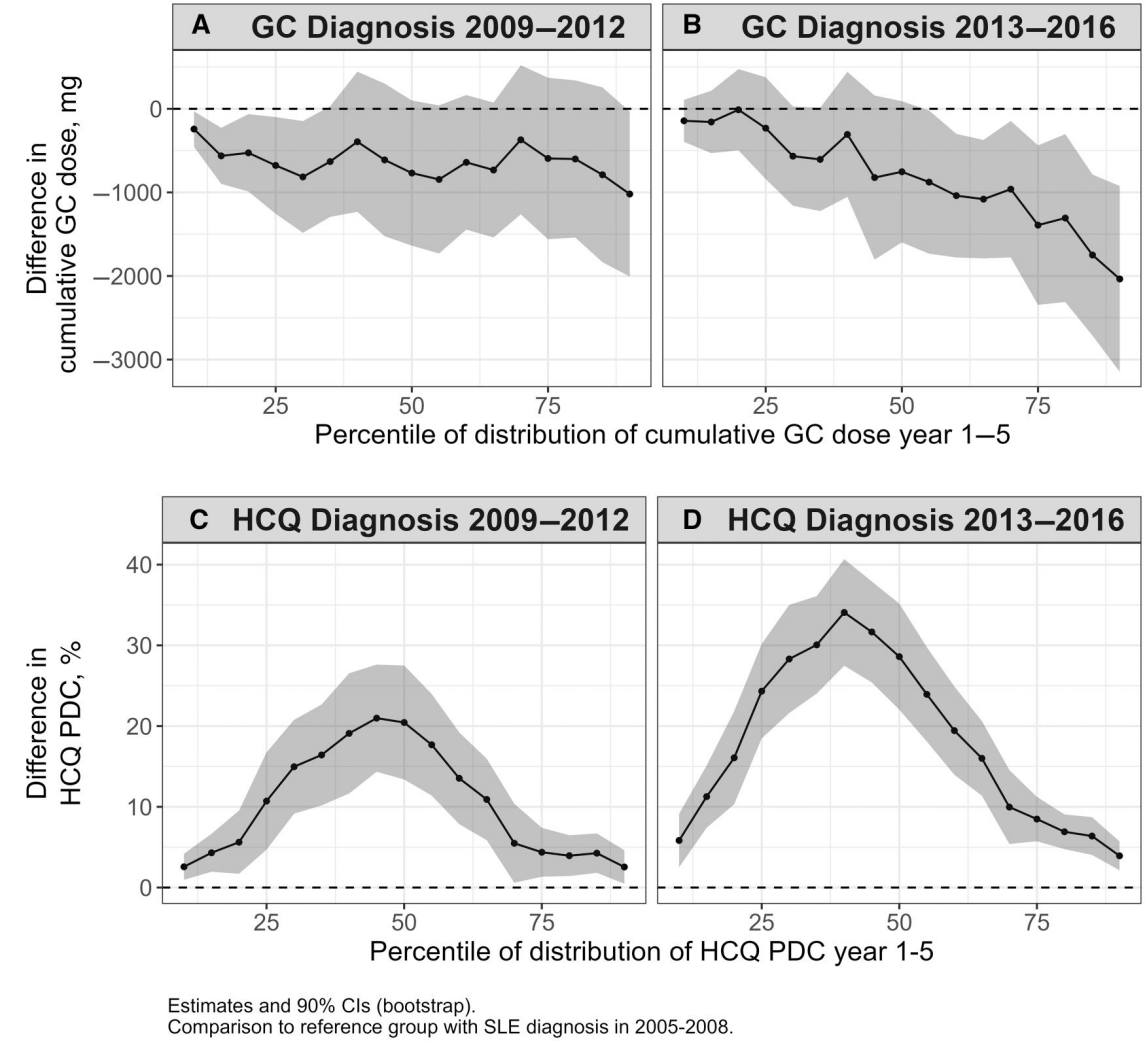
Results from quantile regression of cumulative glucocorticoid (GC) dose (**A** and **B**), and hydroxychloroquine (HCQ) proportion of days covered (PDC; **C** and **D**), over the first 5 years after SLE diagnosis. The difference on the *y*-axis for each percentile of the distribution of 5-year exposure on the *x*-axis is the estimated difference attributable to year of diagnosis (adjusted for sex, age, educational level, country of birth, hospital where receiving the SLE diagnosis, and health care utilization). It should be interpreted in relation to the 5-year exposure distribution in the reference cohort with SLE diagnosis 2005–2008. For example, the decrease in median (50th percentile) for the 2013–2016 cohort of 753 mg (**B**) should be compared with the median 5-year exposure of 7500 mg in the reference cohort with diagnosis in 2005–2008. The increase in median HCQ coverage of 28.6% of days (**D**) should be compared with HCQ 5-year coverage of 50.2% in the reference cohort

In quantile regression analyses of GC cumulative dose over the first 5 years, adjusted for patient characteristics, the decrease in median cumulative dose attributable to calendar year was 769 mg (s.e. 528 mg) and 753 mg (s.e. 514 mg) for 2009–2012 and 2013–2016, respectively, compared with 2005–2008 (Fig. 3). This suggests that especially high GC cumulative doses have decreased with increasing year of diagnosis (percentiles to the right on the *x*-axis) in the most recent years. High levels of cumulative GC dose decreased with older age (Supplementary Fig. S1D, available at *Rheumatology* online). Receiving the SLE diagnosis at a university hospital (Supplementary Fig. S1H) was in general associated with higher GC cumulative doses, except for dose levels in the upper quartile of the distribution, which were less likely for patients with the SLE diagnosis from a university hospital. Increased number of hospitalization days was associated with high cumulative GC doses across the full distribution (Supplementary Fig. S1I and J).

Among patients treated with HCQ at least once during the first 5 years after diagnosis, the median (and interquartile range) HCQ PDC was 50.2% (16.5–80.6%), 66.8% (25.1–85.8%), and 76.9% (42.2–88.5%) for diagnosis years 2005–2008, 2009–2012 and 2013–2016, respectively (*P* < 0.001 for all Wilcoxon two-sample tests for group comparisons).

Results from quantile regression of HCQ coverage are shown in Fig. 3. Compared with diagnosis 2005–2008, the median 5-year PDC was 20.4% (s.e. 4.3%) higher for diagnosis year 2009–2012 (Fig. 3C) and 28.6% (s.e. 4.0%) higher for diagnosis year 2013–2016 (Fig. 3D) conditional on other covariates. Except for the increase of HCQ PDC with calendar time, the most pronounced associations with a high HCQ PDC were high educational level, Nordic country of birth, and SLE diagnosis at university hospital (Supplementary Fig. S2F–H, available at *Rheumatology* online).

### Temporal trends in treatment combinations

Mosaic plots of GC cumulative dose and HCQ PDC over the first 5 years after SLE diagnosis suggest no strong association between cumulative GC average daily dose and HCQ PDC, with patterns similar for the different calendar cohorts (Supplementary Fig. S3, available at *Rheumatology* online). In terms of combinations of treatments, including also IS and summarized yearly, the most common treatment the first year after diagnosis was HCQ+GC (24.7%), followed by IS+HCQ+GC (22.3%), with monotherapy more common later on; the fifth year the most common SLE treatments were HCQ (16.1%) and HCQ+GC (14.8%) (Supplementary Table S4, available at *Rheumatology* online). Alluvial plots stratified on calendar cohort suggest that the increase with calendar time of patients on HCQ comes with an increased proportion also having an IS, especially the first year after diagnosis (Supplementary Fig. S4, available at *Rheumatology* online). Regarding IS, the most common treatments were AZA, MTX and MMF, with a trend towards decreased use of AZA and increased use of MMF (Supplementary Fig. S5, available at *Rheumatology* online).

## Discussion

In this nationwide inception cohort including 3891 SLE patients diagnosed 2005–2021, we showed that the proportion of patients taking oral GC the first year after diagnosis remains high, without any indication of a decrease with calendar time. However, with respect to treatment over the first 5 years after diagnosis we demonstrated some changes in clinical practices with a trend towards tapering of the dose, and more cases of discontinuation of GC, which is in alignment with updated recommendations. Specifically, the proportion of patients on GC 5 years after SLE diagnosis has decreased from 54.1% to 46.3%, which is statistically significant although clinically still judged to be high given the increased risk of organ damage related to GC exposure. The tapering of GC dose is mainly seen in the initial stage after SLE diagnosis from the first to second year after diagnosis. That GC use decreases with follow-up year has been reported earlier [10, 14], although reports of an increase with year of follow-up after diagnosis also exist [8].

In detailed evaluations of the cumulative exposure over the first 5 years after diagnosis, we observed a decrease of the very high cumulative GC doses. To our knowledge this temporal trend towards a decrease of long-term GC exposure has not been shown earlier. In a study of the SLICC inception cohort, GC use was not reduced with increasing year of diagnosis [14]; however, that study was based on data prior to the 2019 update of treatment recommendations, in which the importance of GC limitation was further stressed.

The associations observed between GC exposure and a number of clinical characteristics is in line with what have been observed in previous studies. A decrease of GC exposure with older age has been reported previously [14], and a higher starting dose has been reported for children compared with adults [6, 18]. We observed a trend towards higher levels of cumulative GC doses for patients diagnosed at a university hospital also after adjusting for various patient characteristics. A centre difference was also reported for the SLICC inception cohort [14]. A study of treatment patterns in New Zealand of prevalent SLE showed a discrepancy in treatments by gender, ethnicity, age and socioeconomic factors [19].

The substantial increase in HCQ use in SLE that we observed in terms of both treatment initiation and HCQ coverage is in line with previous reports from other countries [19]. However, we did not see a clear pattern of HCQ being glucocorticoid-sparing. Most patients in our Swedish SLE cohort receive a combination of treatments, which is in line with earlier studies in Asia [20] and the USA [21].

The strength of the current study is that it offers a comprehensive evaluation of temporal trends in the cumulative GC dose, and HCQ coverage, accounting for changes in patient characteristics over time that could have influenced treatment decisions. It is based on national registers and on the amount of medication bought from the pharmacy by the patients and hence represents the medication use outside hospitals in the overall SLE population in Sweden.

A limitation of the current study is that the identification of the study cohort of incident SLE cases relies on ICD codes. Further, we lack data on disease activity, which of course influences the choice of treatment. We examined the possibility of using disease phenotypes such as lupus nephritis as a proxy for disease severity/activity. However, although the occurrence of lupus nephritis was stable over time, it was clear that it was under-reported when identified based on ICD codes from the national registers. Therefore, we adjusted models for number of hospitalization days and specialized outpatient visits as proxies for disease severity and activity that may impact treatment decisions. We were not able to analyse the complete set of SLE treatments due to a lack information on biologic treatments. Further, data on filled prescriptions are only a proxy for consumed medication, though it is unlikely that a patient gets a new dispensation until the previous is consumed. Also, without solid information on prescribed dose regimen, a granular assessment of dose per day could not be performed. Further, our evaluation of GC did not include infusions and medications received at hospitals. Consequently, we do not know if total GC exposure, independent of route of administration, has decreased to the same extent as oral GC use. Also, treatments prior to the first record of an ICD code for SLE are not included in our analysis, and hence patients may in reality have had higher levels of total cumulative doses. Patients in the study for whom 5-year exposure is available had their SLE diagnosis in 2016 at the latest. Consequently, their cumulative 5-year exposure is a mix of prescriptions before and after the updated treatment recommendations were published (in 2019 and 2023). Longer follow-up is needed before the impact of updated recommendations can be fully evaluated.

The association between GC cumulative dose and HCQ coverage and patient characteristics such as sex, age, country of birth and health care utilization may be explained by differences in disease activity, comorbidities and adverse outcomes influencing the choice of treatment over time, aspects that we do not specifically evaluate in this study. However, the association between high HCQ coverage and high educational level, and receiving the SLE diagnosis at a university hospital, motivates continued educational efforts towards raising the awareness of updated management guidelines, towards both treating physicians and patients, to ensure equal care.

GC exposure is known to contribute to irreversible organ damage, including overall cardiovascular events, osteoporosis with fractures and osteonecrosis [11]. Quantification of the reduction of organ damage and mortality as a consequence of improved disease management was out of scope for this study. However, organ damage in our nationwide cohort has not improved over the study time period [22], and neither has mortality (5-year mortality estimated to be 9.5%, 9.7% and 9.3% for cohorts with SLE diagnosis in 2005–2008, 2009–2012 and 2013–2016, respectively [unpublished results]). Globally, a trend of decreased mortality in SLE was seen from the 1950s to 1980s, but it has not improved much since then [23, 24].

The observed reduction of GC use is marginal. The median decrease in the 5-year cumulative GC dose over the course of the study period was only 753 mg, corresponding to a 0.4 mg decrease in GC daily dose over 5 years per patient. Previous studies suggest that this may have a small effect on adverse outcomes; for example it has been estimated that a 1 mg/day decrease in average daily dose would decrease the risk of organ damage by 3% [25]. In another study, only a very high cumulative dose (>560 mg/month) came with a clear increased risk of organ damage [26]. In contrast to GC, HCQ use increased substantially with a median increase in HCQ PDC of 28.6% over the course of the study. Further research on the long-term usage of GC and HCQ, and how SLE management can be optimized, considering other treatment alternatives available, to improve clinical outcomes remains critically important.

## Supplementary Material

keaf192_Supplementary_Data

## Data Availability

It is not possible to publicly share the individual-level data used in this project due to the legal framework governing the raw data. For requests for study data, please contact the corresponding author.
