# Supplementary material for: Temporal trends in glucocorticoids and hydroxychloroquine for treatment of systemic lupus erythematosus in Sweden
Source: Rheumatology (Oxford). 2025 Apr 10;64(8):4622–30. doi: 10.1093/rheumatology/keaf192 (PMC12316358; doi:10.1093/rheumatology/keaf192)
Supplement: keaf192_Supplementary_Data [file keaf192_supplementary_data.docx]

# Supplementary material

Supplementary Table S1 Definitions of variables.

| **Variable** | **Definition** |
| --- | --- |
| SLE treatments | Identification based on Anatomical Therapeutic Chemical Code (ATC)  Glucocorticoids: H02AB Hydroxychloroquine: P01BA02 Azathioprine: L04AX01 Methotrexate: L01BA and L04AX03 Mycophenolate mofetil: L04AA06 Cyclophosphamide: L01AA01 Leflunomide: L04AA13 Tacrolimus: L04AD02 Cyclosporine: L04AD01 |
| Glucocorticoid dose  (prednisolone equivalent dose) | Transformation factors for glucocorticoids to prednisolone equivalent dose:  Cortisone 0.2 Hydrocortisone 0.25 Prednisone 1 Prednisolone 1 Methylprednisolone 1.25 Betamethasone 8.3 Dexamethasone 6.7 |
| University hospitals | Akademiska sjukhuset Uppsala,  Karolinska Universitetssjukhuset Solna,  Karolinska Universitetssjukhuset Huddinge,  Nya Karolinska Solna,  Norrlands universitetssjukhus, Umeå,  Sahlgrenska Universitetssjukhuset, Göteborg,  Skånes universitetssjukhus Malmö,  Skånes universitetssjukhus Lund,  Universitetssjukhuset i Linköping,  Universitetssjukhuset, Örebro |

Supplementary Table S2. Association between glucocorticoid use 1^st^ and 5^th^ year after SLE diagnosis and patient characteristics.

|  |  | Year 1 | | Year 5 | |
| --- | --- | --- | --- | --- | --- |
|  |  | Unadjusted  OR (95%CI) | Adjusted OR (95%CI) | Unadjusted OR (95%CI) | Adjusted OR (95%CI) |
| Year of diagnosis | 2005-2008 | - | - | - | - |
|  | 2009-2012 | 1.28 (1.04-1.56, p=0.018) | 1.23 (1.00-1.52, p=0.048) | 0.83 (0.69-1.01, p=0.064) | 0.81 (0.66-0.98, p=0.032) |
|  | 2013-2016 | 1.28 (1.04-1.57, p=0.018) | 1.21 (0.98-1.50, p=0.070) | 0.73 (0.60-0.89, p=0.001) | 0.71 (0.58-0.86, p=0.001) |
|  | 2017-2021 | 1.01 (0.83-1.23, p=0.904) | 0.98 (0.80-1.21, p=0.862) | - | - |
| Sex | Female | - | - | - | - |
|  | Male | 1.28 (1.06-1.55, p=0.011) | 1.22 (1.00-1.49, p=0.056) | 1.11 (0.90-1.37, p=0.337) | 1.02 (0.82-1.27, p=0.846) |
| Age SLE diagnosis |  | 1.00 (0.99-1.00, p=0.069) | 0.99 (0.99-1.00, p=0.001) | 1.01 (1.00-1.01, p=0.007) | 1.00 (1.00-1.01, p=0.065) |
| Educational level | 0-9y | - | - | - | - |
|  | 10-12y | 1.05 (0.88-1.27, p=0.572) | 1.09 (0.90-1.32, p=0.385) | 1.02 (0.84-1.25, p=0.826) | 1.09 (0.88-1.34, p=0.438) |
|  | ≥13y | 0.88 (0.73-1.06, p=0.187) | 0.94 (0.77-1.15, p=0.544) | 0.85 (0.68-1.05, p=0.124) | 0.96 (0.77-1.20, p=0.702) |
| Country of birth | Nordic | - | - | - | - |
|  | Non-Nordic | 1.27 (1.04-1.55, p=0.019) | 1.21 (0.98-1.49, p=0.085) | 1.10 (0.89-1.37, p=0.374) | 1.20 (0.96-1.51, p=0.116) |
| SLE diagnosis | University hospital | - | - | - | - |
|  | Other hospital | 0.91 (0.79-1.04, p=0.169) | 1.02 (0.88-1.18, p=0.797) | 1.08 (0.92-1.26, p=0.348) | 1.11 (0.94-1.30, p=0.214) |
| Hospitalisation days* | 0 | - | - | - | - |
|  | 1-7 | 1.75 (1.45-2.11, p<0.001) | 1.60 (1.31-1.94, p<0.001) | 1.29 (1.06-1.57, p=0.010) | 1.25 (1.02-1.54, p=0.029) |
|  | >7 | 2.68 (2.15-3.36, p<0.001) | 2.58 (2.04-3.30, p<0.001) | 1.67 (1.34-2.08, p<0.001) | 1.53 (1.21-1.93, p<0.001) |
| Outpatient visits* | 0-2 | - | - | - | - |
|  | 3-6 | 1.21 (1.03-1.42, p=0.021) | 1.07 (0.90-1.27, p=0.442) | 1.16 (0.97-1.38, p=0.115) | 1.08 (0.90-1.30, p=0.404) |
|  | >6 | 1.61 (1.35-1.92, p<0.001) | 1.28 (1.05-1.55, p=0.014) | 1.43 (1.17-1.74, p<0.001) | 1.30 (1.05-1.60, p=0.015) |

Odds ratios (OR) and 95% confidence intervals (95%CI) from univariable and multivariable logistic regression analyses of having at least one glucocorticoid dispensation during 1^st^ and 5^th^ year after SLE diagnosis.

*within 1 year preceding SLE diagnosis.

SLE: Systemic lupus erythematosus

Supplementary Table S3. Association between hydroxychloroquine use 1^st^ and 5^th^ year after SLE diagnosis and patient characteristics.

|  |  | Year 1 | | Year 5 | |
| --- | --- | --- | --- | --- | --- |
|  |  | Unadjusted OR (95%CI) | Adjusted OR (95%CI) | Unadjusted OR (95%CI) | Adjusted OR (95%CI) |
| Year of diagnosis | 2005-2008 | - | - | - | - |
|  | 2009-2012 | 2.24 (1.85-2.70, p<0.001) | 2.46 (2.02-3.00, p<0.001) | 1.53 (1.26-1.85, p<0.001) | 1.61 (1.32-1.96, p<0.001) |
|  | 2013-2016 | 3.42 (2.81-4.16, p<0.001) | 3.90 (3.16-4.82, p<0.001) | 2.19 (1.80-2.67, p<0.001) | 2.32 (1.89-2.85, p<0.001) |
|  | 2017-2021 | 5.10 (4.18-6.25, p<0.001) | 5.53 (4.44-6.91, p<0.001) | - | - |
| Sex | Female | - | - | - | - |
|  | Male | 0.80 (0.67-0.94, p=0.009) | 1.05 (0.87-1.28, p=0.619) | 1.00 (0.81-1.24, p=0.985) | 1.20 (0.96-1.50, p=0.110) |
| Age SLE diagnosis | Mean (SD) | 0.97 (0.97-0.98, p<0.001) | 0.97 (0.97-0.98, p<0.001) | 0.98 (0.97-0.98, p<0.001) | 0.98 (0.97-0.98, p<0.001) |
| Educational level | 0-9y | - | - | - | - |
|  | 10-12y | 1.36 (1.14-1.62, p<0.001) | 1.03 (0.85-1.24, p=0.776) | 1.29 (1.05-1.58, p=0.013) | 1.14 (0.92-1.41, p=0.234) |
|  | ≥13y | 1.50 (1.25-1.80, p<0.001) | 0.96 (0.78-1.18, p=0.712) | 1.42 (1.15-1.76, p=0.001) | 1.15 (0.92-1.45, p=0.219) |
| Country of birth | Nordic | - | - | - | - |
|  | Non-Nordic | 1.57 (1.29-1.91, p<0.001) | 1.05 (0.84-1.30, p=0.688) | 1.26 (1.02-1.57, p=0.034) | 1.00 (0.79-1.26, p=0.997) |
| SLE diagnosis | University hospital | - | - | - | - |
|  | Other hospital | 0.80 (0.70-0.92, p=0.001) | 0.84 (0.73-0.98, p=0.026) | 0.74 (0.64-0.87, p<0.001) | 0.80 (0.68-0.95, p=0.009) |
| Hospitalisation days* | 0 | - | - | - | - |
|  | 1-7 | 0.82 (0.69-0.97, p=0.019) | 0.86 (0.71-1.05, p=0.137) | 0.93 (0.76-1.13, p=0.445) | 0.94 (0.76-1.16, p=0.558) |
|  | >7 | 0.58 (0.49-0.70, p<0.001) | 0.77 (0.63-0.95, p=0.012) | 0.84 (0.68-1.04, p=0.107) | 0.97 (0.77-1.23, p=0.813) |
| Outpatient visits* | 0-2 | - | - | - | - |
|  | 3-6 | 1.03 (0.88-1.21, p=0.709) | 1.08 (0.90-1.29, p=0.402) | 0.93 (0.78-1.11, p=0.427) | 0.96 (0.79-1.16, p=0.663) |
|  | >6 | 0.79 (0.67-0.94, p=0.006) | 0.75 (0.62-0.92, p=0.004) | 0.83 (0.68-1.01, p=0.063) | 0.78 (0.63-0.96, p=0.021) |

Odds ratios (OR) and 95% confidence intervals (95%CI) from univariable and multivariable logistic regression analyses of having at least one hydroxychloroquine dispensation during 1^st^ and 5^th^ year after SLE diagnosis.*within 1 year preceding SLE diagnosis.
SLE: Systemic lupus erythematosus

Supplementary Table S4 Treatment combinations among SLE patients first 5 years after diagnosis.

| **Treatment combination** | Year 1 (N=2794) | Year 2 (N=2740) | Year 3 (N=2690) | Year 4 (N=2639) | Year 5 (N=2587) |
| --- | --- | --- | --- | --- | --- |
|  |  |  |  |  |  |
| IS+GC+HCQ | 623 (22.3%) | 502 (18.3%) | 408 (15.2%) | 390 (14.8%) | 355 (13.7%) |
| IS+GC | 388 (13.9%) | 372 (13.6%) | 336 (12.5%) | 288 (10.9%) | 252 (9.7%) |
| IS+HCQ | 50 (1.8%) | 116 (4.2%) | 158 (5.9%) | 171 (6.5%) | 169 (6.5%) |
| IS only | 48 (1.7%) | 83 (3.0%) | 124 (4.6%) | 115 (4.4%) | 103 (4.0%) |
| GC+HCQ | 691 (24.7%) | 475 (17.3%) | 425 (15.8%) | 398 (15.1%) | 384 (14.8%) |
| GC only | 297 (10.6%) | 321 (11.7%) | 300 (11.2%) | 314 (11.9%) | 298 (11.5%) |
| HCQ only | 345 (12.3%) | 378 (13.8%) | 413 (15.4%) | 402 (15.2%) | 416 (16.1%) |
| Other* | 352 (12.6%) | 493 (18.0%) | 526 (19.6%) | 561 (21.3%) | 610 (23.6%) |

GC: Glucocorticoid; HCQ: Hydroxychloroquine; IS: Immunosuppressive drug
* Includes patients with other treatment or no treatment.


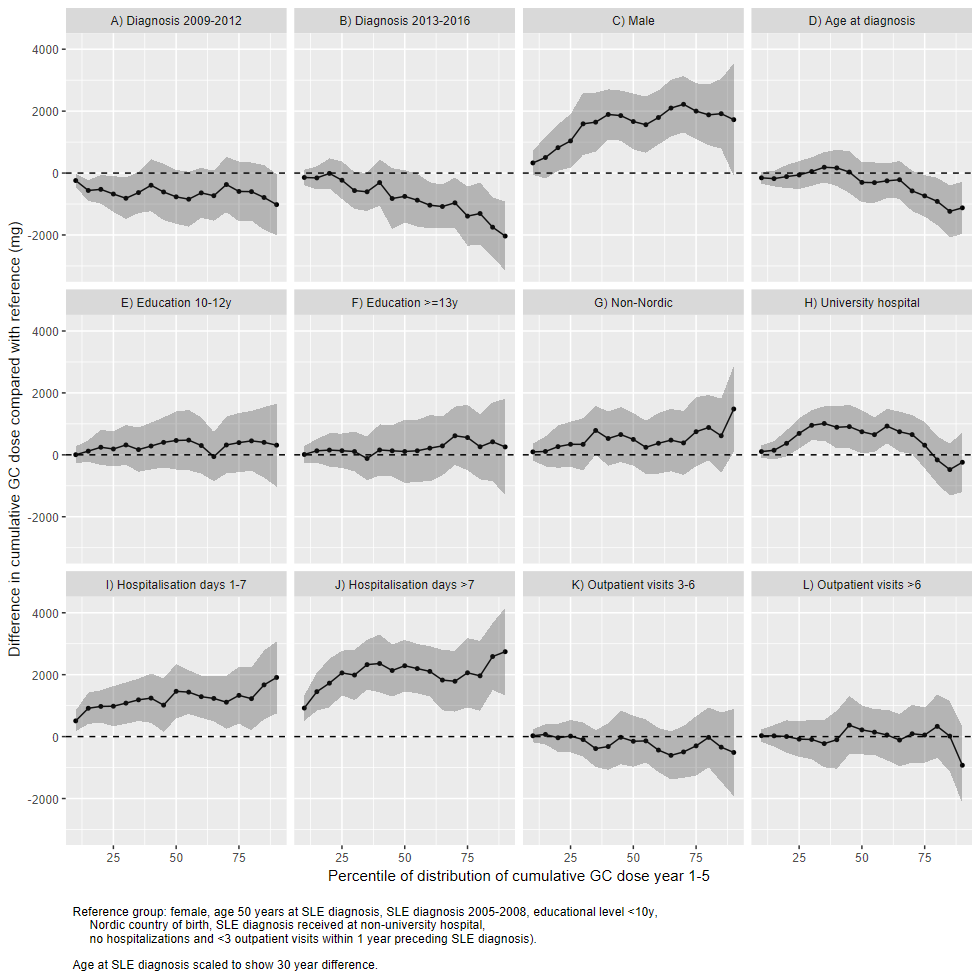


Supplementary Figure S1 Difference in cumulative glucocorticoid (GC) dose over the first 5 years after SLE diagnosis, estimated from quantile regression. The difference on the y-axis is the estimated difference attributable to each factor (conditional on other factors) compared to the reference level, with an estimated difference for each percentile of the distribution of 5-year exposure on the x-axis. Reference group: female, age 50 at diagnosis, diagnosed 2005-2008, educational level <10 years, Nordic country of birth, SLE diagnosis at non-university hospital, no hospitalisation and <3 outpatient visits during 1 year preceding SLE diagnosis.


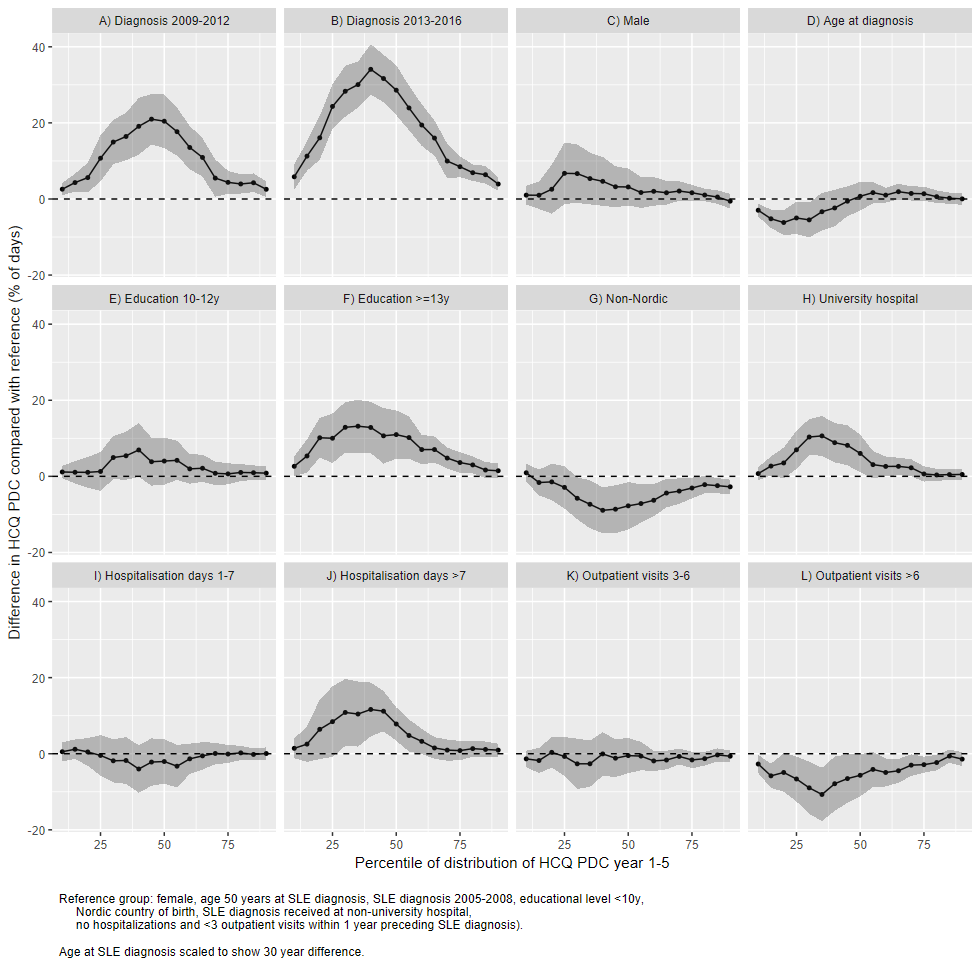


Supplementary Figure S2 Difference in hydroxychloroquine (HCQ) proportion of days covered (PDC) over the first 5 years after SLE diagnosis, estimated from quantile regression. The difference on the y-axis is the estimated difference attributable to each factor (conditional on other factors) compared to the reference level, with an estimated difference for each percentile of the distribution of 5-year exposure on the x-axis. Reference group: female, age 50 at diagnosis, diagnosed 2005-2008, educational level <10 years, Nordic country of birth, SLE diagnosis at non-university hospital, no hospitalization and <3 outpatient visits during 1 year preceding SLE diagnosis.


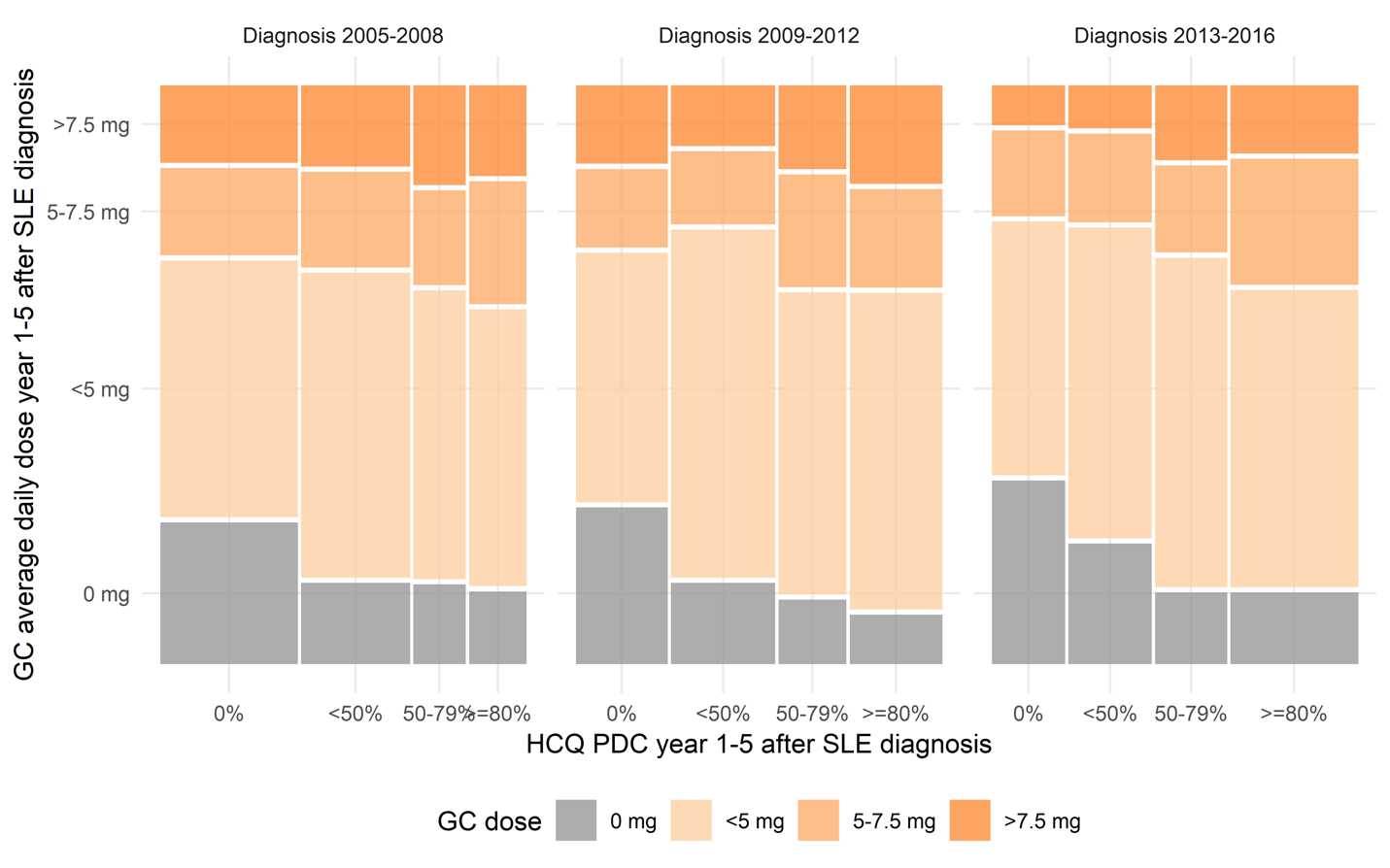


*Supplementary Figure S3 Relationship between glucocorticoid average daily dose and hydroxychloroquine proportion of days covered summarized over first 5 years after SLE diagnosis.*


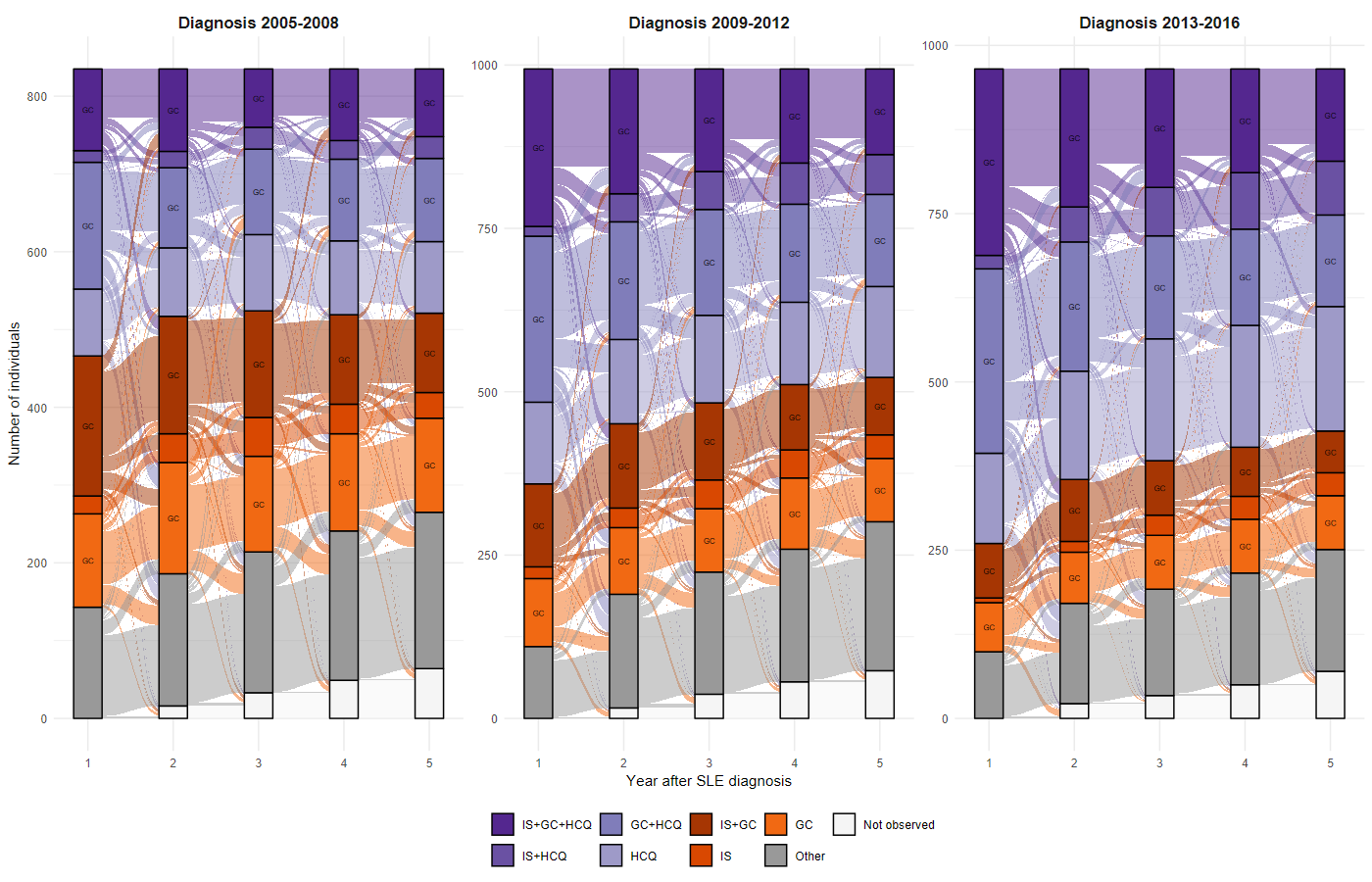


Supplementary Figure S4 Combination of SLE treatments first 5 years after SLE diagnosis.


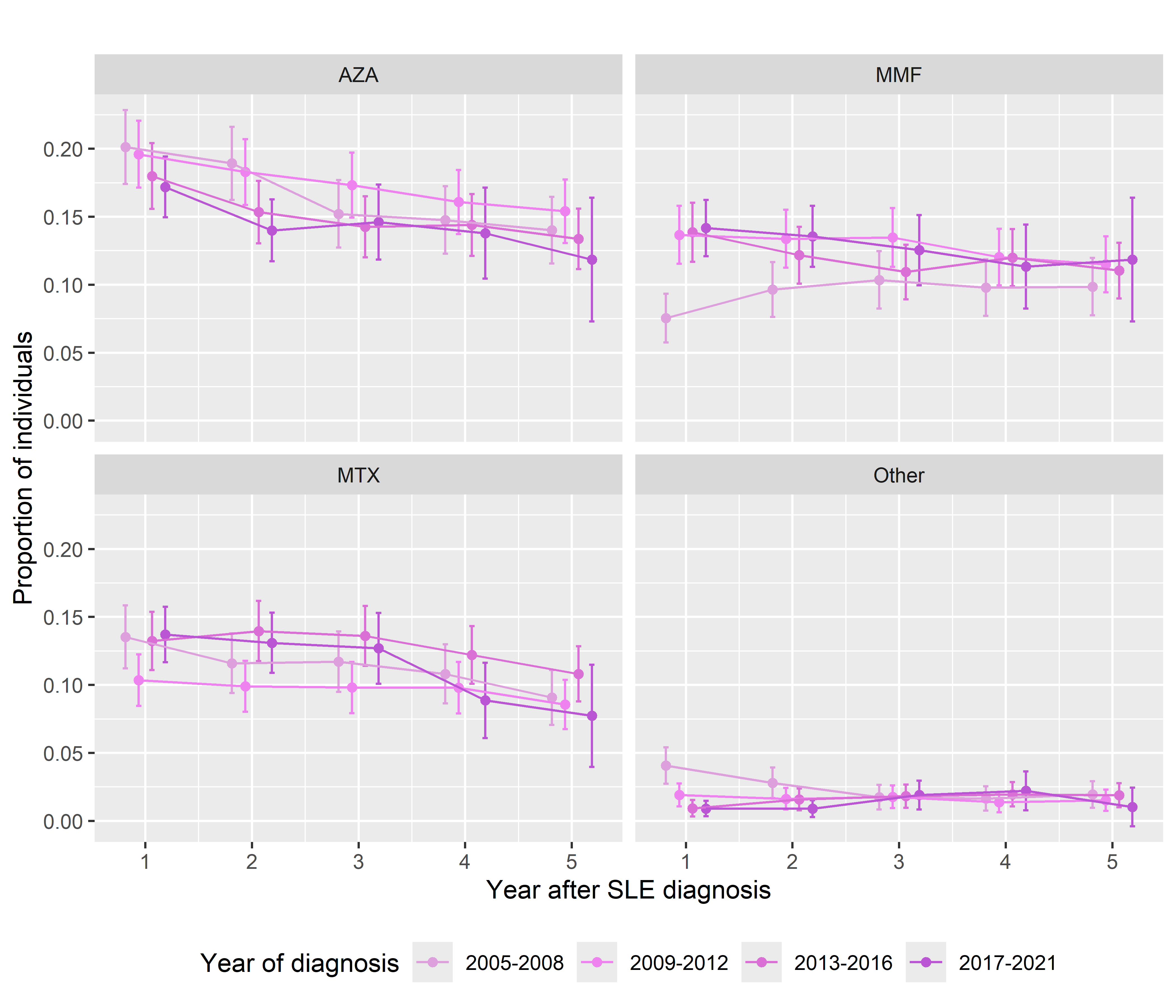


Supplementary Figure S5 Proportion of individuals with systemic lupus erythematosus with at least one dispensation of the most frequently prescribed disease-modifying antirheumatic drugs, including azathioprine (AZA), mycophenolate mofetil (MMF), and methotrexate (MTX), evaluated annually.
